# Effect of supplementation with probiotics or synbiotics on cardiovascular risk factors in patients with metabolic syndrome: a systematic review and meta-analysis of randomized clinical trials

**DOI:** 10.3389/fendo.2023.1282699

**Published:** 2024-01-08

**Authors:** TingRui Chen, Jing Wang, ZeKun Liu, Fei Gao

**Affiliations:** Department of Endocrinology, First Hospital of Shanxi Medical University, Taiyuan, Shanxi, China

**Keywords:** probiotics, synbiotics, metabolic syndrome, cardiovascular, systematic review

## Abstract

**Purpose:**

The effectiveness of probiotics or synbiotics in adults with metabolic syndromes (MetS) remains controversial, this meta-analysis will further analyze the effects of probiotics or synbiotics on cardiovascular factors in adults with MetS.

**Methods:**

We searched Web of Science, PubMed, Embase, Cochrane Library and other databases for randomized controlled trials (RCTs) on the effects of probiotics or synbiotics on MetS in adults up to July 2023, and used RevMan 5.4.0 software for statistical analysis.

**Results:**

This analysis included eleven RCTs (n = 608 participants), and the results showed that compared with the control group, supplementation with probiotics or synbiotics reduced body mass index (weighted mean difference, WMD = -0.83, 95% CI = [-1.21, -0.44], P <0.0001, n = 9), low-density lipoprotein (LDL-c) (standard mean difference, SMD = -0.24, 95% CI = [-0.41, -0.08], P = 0.004, n = 10), fasting blood glucose (FBG)(SMD = -0.17, 95% CI = [-0.33, -0.01], P = 0.03, n = 11), but had no beneficial effect on systolic blood pressure (SBP) (WMD = 1.24, 95% CI = [-2.06, 4.54], P = 0.46, n = 8) in MetS patients.

**Conclusion:**

Supplementation with probiotics or synbiotics can reduce BMI, LDL-c, FBG in patients with MetS, but our findings did not demonstrate a favorable effect on reducing SBP. Future studies with larger samples and longer intervention periods are needed.

## 1 Introduction

Metabolic syndrome (MetS) is a multifactorial metabolic syndrome (1), which is mainly manifested as insulin resistance, dyslipidemia, hypertension, and central obesity (2). Abnormal metabolism in patients with MetS significantly increases the prevalence of cardiovascular disease and type 2 diabetes (3). It is estimated that the prevalence of cardiovascular disease (CVD) in patients with MetS is about twice that in the general population, and the prevalence of type 2 diabetes is approximately five times higher than that in the general population (4, 5). With the rapid development of the economy and the improvement of living standards, the prevalence of MetS has shown a significant upward trend globally, and its average prevalence in the world is about 20-25% (6), which has become a major public health challenge. How to control the abnormal metabolism of MetS patients is particularly important, however, the options for drugs that can effectively improve the overall abnormal metabolism of MetS patients are very limited.

There are relevant literature reports (7), the imbalance of intestinal flora is closely related to the clinical manifestations of patients with MetS (6, 8–10), probiotics are often used to improve host gut microbiota homeostasis (11), and exert positive effects on the body by regulating the host gut microbiota (12). In recent years, more and more studies have begun to focus on the role of probiotics in metabolic syndrome (13, 14), some studies have found that probiotic supplementation is effective in improving MetS (15, 16), while others have found no relationship (17). In order to further understand the relationship between the two, this article incorporated the latest randomized controlled trials for retrospective study, providing valuable evidence-based science for clinical work.

## 2 Materials and methods

### 2.1 Literature sources and search methods

This meta-analysis was conducted according to the preferred reporting items of the systematic review and meta-analysis (PRISMA) guidelines (18). An author independently searched the Web of Science, PubMed, Embase, Cochrane Library and other databases. The search period is from the establishment of the library to July 2023. The combination of subject words and free text terms was used to ensure the recall and precision of the retrieval. The search terms were as follows: “metabolic syndrome*”, “syndrome*, metabolic”, “insulin resistance syndrome*”, “syndrome*, “insulin resistance”, “dysmetabolic syndrome*”, “reaven syndrome*”, “syndrome*, reaven”, “cardiometabolic syndrome*”, “syndrome*, cardiometabolic”, “cardiometabolic syndrome*, metabolic”, “metabolic cardiometabolic syndrome*”, “prebiotic*”, “probiotic*”, “synbiotic*”, and searched by logical operations.

### 2.2 Inclusion and exclusion criteria

The selection criteria were as follows: (1) Participants: Patients with metabolic syndrome who meet any of the diagnostic criteria, regardless of gender and course of disease, aged ≥ 18 years; (2) Intervention: Participants in the experimental group were treated with probiotics, synbiotics or dairy products containing probiotics; (3) The control group used placebo or plain yogurt as a control; (4) Outcome measures: Report at least one of the following outcomes: body mass index (BMI), low-density lipoprotein (LDL-c), fasting blood glucose (FBG), systolic blood pressure (SBP); (5) Study type: All included studies were randomized controlled trials, language is not limited.

Exclusion criteria were as follows: (1) duplicate published articles; (2) systematic reviews, review articles, conference abstracts, (3) non-randomized controlled trials; (4) articles with inconsistent interventions, subjects, and no corresponding outcome measures.

### 2.3 Data extraction

The two authors (C-TR and W.J.) independently extracted the following information from the full text, including first author, year of publication, country, diagnosis, age, sample size of the intervention group, sample size of the control group, course of medication, dose of medication, and reported outcome indicators. The data of outcome indicators in some studies were expressed in the form of median and quartile or mean and standard error, which were uniformly converted into mean and standard deviation (19, 20).

### 2.4 Quality assessment

The included studies were assessed for quality according to RoB 1.0 (21) in terms of randomization sequence generation, allocation concealment, blinding of participants and experimental personnel, blinding of outcome assessors, completeness of outcome data, selective reporting of results, and other bias. The two authors (C-TR and W.J.) independently completed the quality assessment of the literature, and all disagreements were agreed upon after discussion with a third researcher.

### 2.5 Statistical analysis

We used RevMan 5.4.0 software for statistical analysis. Continuous variables were expressed as standard mean difference (SMD) or weighted mean difference (WMD) with 95% confidence interval (95% CI). SMD was used to combine effect sizes when the dosage units of outcome measures were not uniform, and WMD was used otherwise. Cochrane’s Q and I^2^ statistics were used to determine whether there was heterogeneity (22). I^2^ > 50% and P < 0.10 as a threshold indicating significant heterogeneity, analyses should be performed using a random-effects model; I^2^ < 50% and P > 0.10 suggesting less heterogeneity between studies, while a fixed-effect model was used. Sensitivity analyses were conducted by removing the included studies individually. Funnel plots were used to analyze publication bias.

## 3 Results

### 3.1 Literature search

**Figure 1:**
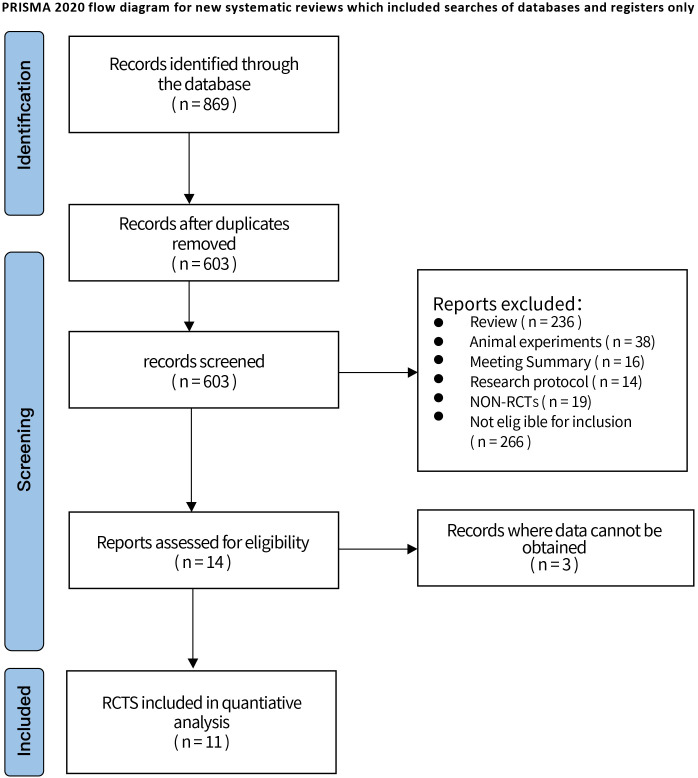
Retrieval flowchart.

Through the search of databases, a total of 869 articles were retrieved, including 395 articles from Web of Science, 103 articles from PubMed, 134 articles from Embase and 237 articles from Cochrane Library. After removing 266 duplicate articles, 603 articles remained; browsing of literature titles and abstracts, 236 reviews and systematic evaluations, 38 animal experiments, 16 meeting summaries, 14 researching protocols, 19 non-RCTs, 266 not eligible for inclusion criteria, 3 articles for which data could not be obtained were excluded. Finally, 11 literatures (23–33) were included, all in English. The PRISMA flow diagram and general characteristics of the included studies are shown in **Figure 1** and **Supplementary Table S1**.

### 3.2 Quality assessment of included studies

**Figure 2:**
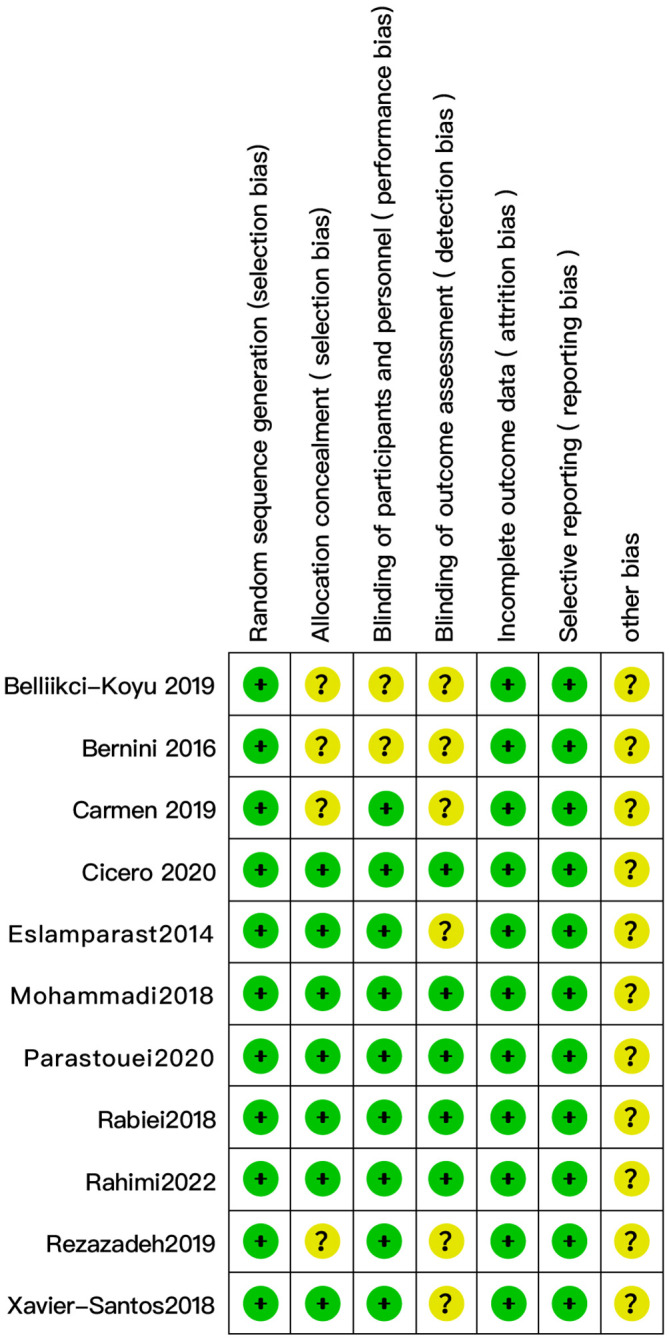
Evaluation of the quality of studies included in the meta-analysis (n = 11).

The quality of the 11 randomized controlled trials included in this review was assessed using RoB 1.0, all of which used random sequence generation. Seven studies had a low risk of the allocation concealment (23–28, 31), and four studies were rated as unclear (29, 30, 32, 33). Participants and personnel were blind in nine trials (23–29, 31, 32), and outcome assessors were blind in five trials (26–29, 31). All trials showed a low risk of bias in domains of incomplete outcomes data and selective reporting (**Figure 2**).

### 3.3 Meta-analysis results

#### 3.3.1 Supplementation with probiotics or synbiotics and body mass index

**Figure 3:**
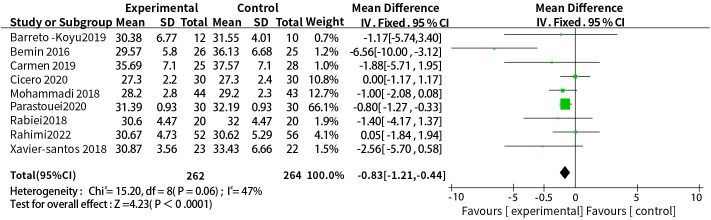
Probiotic or synbiotic supplementation versus BMI in the control group.

A total of nine studies reported the relationship between the supplementation with probiotics or synbiotics and BMI, the results of heterogeneity test were: P = 0.06, I^2^ = 47%, the heterogeneity was relatively small, and the fixed-effect model was used. The final results showed that supplementation with probiotics or synbiotics can effectively control BMI levels (WMD = -0.83, 95% CI = [-1.21, -0.44], P <0.0001, n = 9) (**Figure 3**).

#### 3.3.2 Supplementation with probiotics or synbiotics and low-density lipoprotein

**Figure 4:**
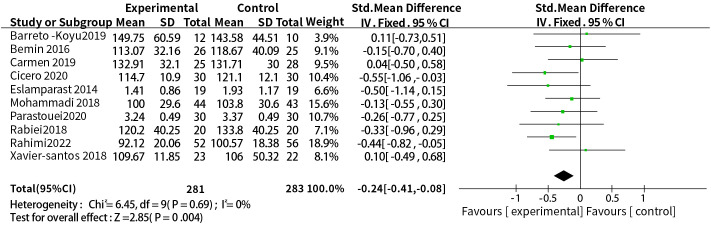
Probiotic or synbiotic supplementation versus LDL-c in the control group.

A total of ten studies reported the effect of probiotic or synbiotic supplementation on LDL-c in people with MetS. The dosage units between the studies were different, so SMD combined effect size was selected, the results of heterogeneity test were P = 0.69, I^2^ = 0%, no heterogeneity was found between studies, so a fixed-effects model was used. The final results showed that supplementation with probiotics or synbiotics reduced LDL-c (SMD = -0.24, 95% CI = [-0.41, -0.08], P = 0.004, n = 10) (**Figure 4**).

#### 3.3.3 Supplementation with probiotics or synbiotics and fasting blood glucose

**Figure 5:**
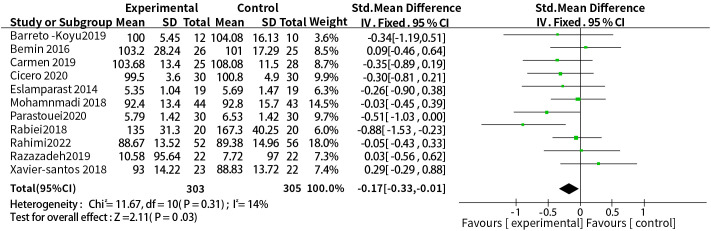
Probiotic or synbiotic supplementation versus FBG in the control group.

A total of eleven studies reported a relationship between the two. The dosage units between studies were different, and the combined effect size of SMD was selected, the results of heterogeneity test were P = 0.31, I^2^ = 14%, so a fixed-effect model was used. The results showed that probiotic or synbiotic supplementation significantly reduced FBG compared to placebo (SMD = -0.17, 95% CI = [-0.33, -0.01], P = 0.03, n = 11) (**Figure 5**).

#### 3.3.4 Supplementation with probiotics or synbiotics and systolic blood pressure

**Figure 6:**
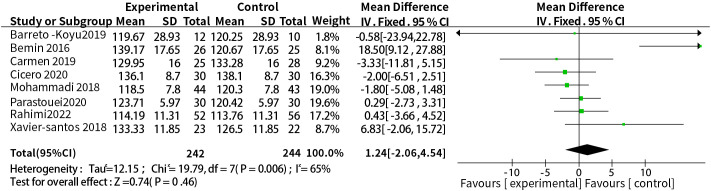
Probiotic or synbiotic supplementation versus SBP in the control group.

**Figure 7:**
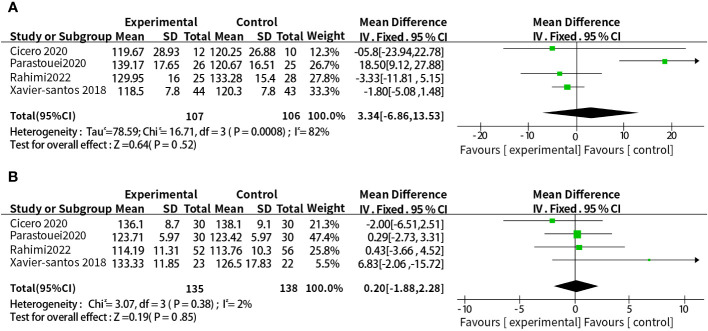
**(A)** Subgroup analysis of the effect of probiotic supplementation alone on SBP. **(B)** Subgroup analysis of the effect of synbiotic supplementation alone on SBP.

A total of eight studies reported the relationship between supplementation with probiotics or synbiotics and SBP. The results of heterogeneity test were: P =0.006, I^2^ = 65%, because of the large heterogeneity, the random-effect model was selected. The results showed that probiotic or synbiotic supplementation did not change SBP compared to placebo (WMD = 1.24, 95% CI = [-2.06, 4.54], P = 0.46, n = 8) (**Figure 6**). Arabi et al. (15) observed the supporting effect of synbiotics on SBP in a systematic review of the effect of synbiotic supplementation in MetS patients in MetS patients, and we analyzed synbiotic supplementation or prebiotic supplementation as subgroups to assess the effect of both on SBP alone, and did not find a supportive effect of probiotic supplementation (WMD = 3.34, 95% CI = [-6.86, 13. 53], P = 0.52, n = 4) or synbiotic supplementation (WMD = 0.20, 95% CI = [-1.88, 2.28], P = 0.85, n = 4) on SBP (**Figure 7**).

### 3.4 Sensitivity analysis and publication bias

**Figure 8:**
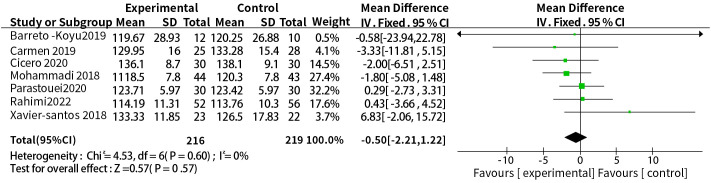
Sensitivity analysis of the effect of probiotic or synbiotic supplementation on SBP.

**Figure 9:**
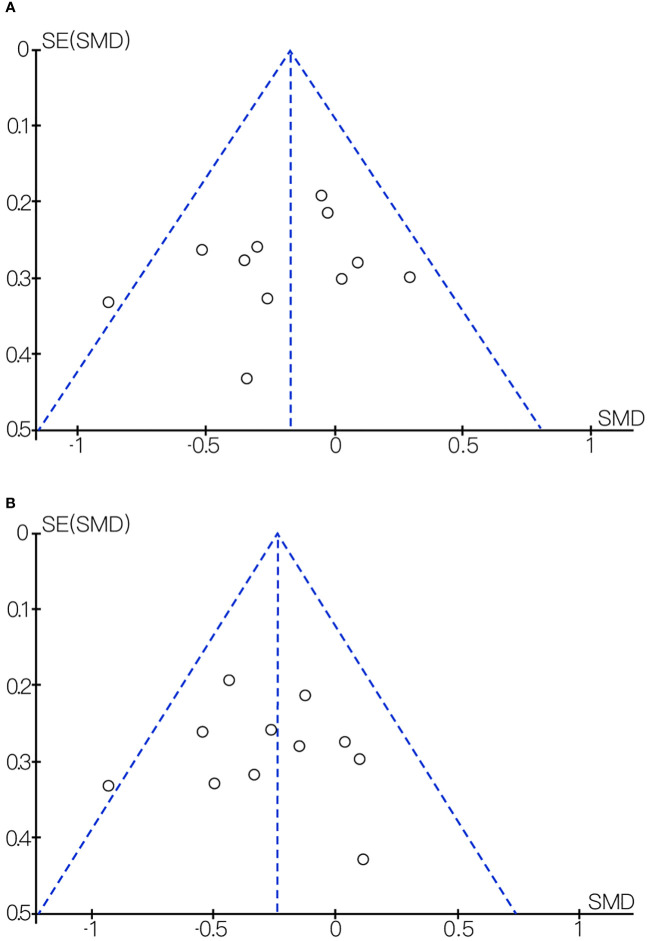
**(A)** Funnel diagram of the effect of probiotic or synbiotic supplementation on FBG. **(B)** Funnel diagram of the effect of probiotic or synbiotic supplementation on LDL-c.

We found considerable heterogeneity in SBP, which affected the accuracy of the results. Through sensitive analysis, we found that Bernini et al. (30) affected the reliability of the SBP results, and the heterogeneity result after excluding this study was: P = 0.60, I^2^ = 0%, after removing the study of Bernini et al. (30), the result indicate the effect of probiotic or synbiotic supplementation on SBP had no significance (WMD = -0.50, 95% CI = [-2.21, 1.22], P = 0.57, n = 7) (**Figure 8**), which may be related to the unclear blinding of participants, experimenters, and outcome assessors in the Bernini et al. (30) study. The funnel plots were drawn by outcome indicators of FBG and LDL-c, and the funnel plots of the two outcome index targets were basically symmetrical, with little possibility of bias, therefore, the conclusion was reliable. The funnel diagram was shown in the **Figure 9**.

## 4 Discussion

This was a study of the effect of supplementation of probiotic or synbiotics on cardiovascular risk factors in patients with MetS, the results of meta-analysis of 11 RCTs showed that probiotic or synbiotic supplementation can effectively reduce BMI, LDL-c and FBG in patients with MetS, but the effect on systolic blood pressure was still questionable.

Obesity, as a common manifestation of MetS, is associated with a variety of metabolic diseases. Studies have found (34, 35) that the body affects the gastrointestinal microorganisms and maintains homeostasis of weight through a variety of mechanisms mediated by bidirectional signaling of the gut-brain axis (GBA). Changes in the gut microbiota are strongly associated with obesity, Studies (36) have reported that probiotics can promote health by remodeling the gut microbiota. By analyzing nine studies, we observed that the use of probiotics was effective in improving BMI in patients with Mets, which was consistent with the conclusions of Li G.Z. et al. (16) and Arabi et al. (15) Therefore, the use of probiotics for MetS can achieve a certain weight loss effect, thereby reducing the occurrence of cardiovascular events.

Cardiovascular disease is the leading cause of morbidity and mortality worldwide, and its pathological basis is atherosclerosis (37), the elevation of low-density lipoprotein level is the primary factor of atherosclerosis (38). Studies have found (39, 40) that probiotics stabilize lipid metabolism by regulating bile acid metabolism, inhibiting cholesterol absorption, and enhancing fecal excretion of cholesterol. Therefore, probiotics play an active role in preventing the occurrence and development of cardiovascular diseases. However, Hadi et al. (17) did not support the effectiveness of probiotics supplementation in reducing LDL-c in patients with MetS after a review of nine RCTs. The meta-results concluded that probiotics can effectively reduce LDL-c in patients with MetS, which was consistent with the studies of G.Z. et al. (16) and Arabi (15).

Insulin resistance is characteristic of abnormal glucose metabolism in patients with MetS, and lowering fasting blood glucose in patients can prevent its occurrence and reduce the incidence of type 2 diabetes (41). The role between probiotics and blood glucose was unknown, but it has been reported that (42) probiotics can stabilize blood glucose by modulating, reducing oxidative stress in pancreatic tissue, or regulating body lipid metabolism. After studying 9 RCTs, we found that probiotics supplementation with dietary exercise can reduce fasting blood glucose in patients with MetS to a certain extent and regulate glucose metabolism in patients. Arabi et al. (15) found that synbiotics supplementation had a positive effect on FBG by dissecting the relationship between synbiotics supplementation and MetS patients. However, after the corresponding meta-analysis by Li G. Z. (16), Hadi et al. (17) for the purpose of supplementing the effect of probiotics on MetS, they did not find that probiotics can reduce fasting blood glucose in MetS patients, which contradicted our results, the small number of included studies, the short duration of the intervention, and the absence of dietary exercise interventions may have been the main reasons for the conflict.

Hypertension is an independent risk factor for cardiovascular disease (43). As an important part of blood pressure, systolic blood pressure plays a major role in the occurrence of coronary heart disease and is the main goal of antihypertensive therapy (44, 45). Arabi et al. (15) analyzed three included RCTs and concluded that synbiotics were effective in lowering blood pressure in patients with Mets. Hadi et al. (46) analyzed three RCTs on the effect of synbiotics on blood pressure in adults, but found that synbiotics use only reduced systolic blood pressure. We analyzed eight randomized controlled trials and found no favorable effect of probiotics on blood pressure in people with Mets. In subgroup analyses, we still did not find any effect of probiotics or synbiotics alone on SBP. Due to the limited number of relevant trials, our availability of reliable results was limited, and more trials were needed to further validate the relationship.

In terms of the results of this study, we found that supplementation of probiotics or synbiotics had significant statistical significance for the improvement of BMI, LDL-c and FBG in Mets patients. This suggests that supplementation with probiotics or synbiotics can effectively reduce BMI, LDL-c and FBG in Mets patients. Therefore, in the future treatment of Mets patients with obesity, CVD and T2DM, supplementation of probiotics or synbiotics can be used as a new treatment method. However, its effect on blood pressure in Mets patients is not clear, so more high-quality studies are needed to explore. Mets is a pathological state involving dyslipidemia, insulin resistance and cardiovascular disease. Patients with Mets often have a variety of complications, among which diabetes is one of the common complications. In recent years, more and more evidences have shown that (47, 48), the occurrence of T2DM is closely related to the composition of intestinal flora. Oral hypoglycemic drugs are commonly used in the treatment of T2DM. Among them, metformin, acarbose, glucagon-like peptide-1 (GLP-1) and other hypoglycemic drugs achieve hypoglycemic effects by changing the structure of the flora, hormone secretion, bile acid metabolism and inflammatory response (49–51). Sodium-glucose cotransporter 2 inhibitor (SGLT-2), as a new class of oral hypoglycemic drugs, also plays an important role in lowering blood pressure, improving bone metabolism and brain function (52). Its hypoglycemic effect is mainly to control blood glucose by reducing the reabsorption of glucose mediated by proximal renal tubules. Bammens B et al. (53)have shown that SGLT-2 can inhibit the abnormal increase of bifidobacteria, improve the composition of abnormal microflora to a certain extent to reduce blood glucose. In summary, hypoglycemic drugs can affect the composition of intestinal flora, change the metabolites of the flora in patients with T2DM, and then improve glucose metabolism in the body. However, there is no basis for the exploration of clinical use of intestinal flora modulators in the adjuvant treatment of T2DM now, so we still need to provide a useful attempt to use intestinal flora as a new intervention point.

Compared with previous studies, this study included more studies, larger sample size, less publication bias, and relatively reliable conclusions. However, the intervention time of randomized trials was less than 3 months, and if probiotics can effectively improve the indicators of Mets patients, it may take longer treatment. Differences in methodology and inconsistencies in the way probiotics intervene may reduce the intensity of this study. Therefore, future studies with larger samples and longer intervention periods are needed. Therefore, future studies with larger samples and longer intervention periods are needed.

## 5 Conclusion

Probiotics or synbiotics can improve the abnormal metabolic state of patients with metabolic syndrome to a certain extent, especially in reducing weight, improve lipid metabolism and glucose metabolism, but our findings did not demonstrate a favorable effect on reducing SBP. It is expected that there will be a larger sample size and more adequate studies for further demonstration.

## Author contributions

TC: Data curation, Investigation, Methodology, Writing – original draft, Writing – review & editing. JW: Data curation, Investigation, Methodology, Writing – review & editing. FG: Funding acquisition, Supervision, Validation, Writing – review & editing. ZL: Writing – original draft.
