# Supplementary material for: Effect of supplementation with probiotics or synbiotics on cardiovascular risk factors in patients with metabolic syndrome: a systematic review and meta-analysis of randomized clinical trials
Source: Front Endocrinol (Lausanne). 2024 Jan 8;14:1282699. doi: 10.3389/fendo.2023.1282699 (PMC10801034; doi:10.3389/fendo.2023.1282699)
Supplement: Supplementary file 1 [file Table_1.docx]

Supplementary Material

# Supplementary Data

Search Formula：

P: Metabolic Syndrome, I:Supplement with probiotics or synbiotics, S:RCT

PubMed

((("Metabolic Syndrome"[Mesh]) OR (((((((((((Metabolic Syndrome*[Title/Abstract]) OR (Syndrome*,Metabolic[Title/Abstract])) OR (Syndrome*,Insulin Resistance[Title/Abstract])) OR (Insulin Resistance Syndrome*[Title/Abstract])) OR (Dysmetabolic Syndrome*[Title/Abstract])) OR (Reaven Syndrome*[Title/Abstract])) OR (Syndrome*,Reaven[Title/Abstract])) OR (Cardiometabolic Syndrome*[Title/Abstract])) OR (Syndrome*,Cardiometabolic[Title/Abstract])) OR (Cardiovascular Syndrome*,Metabolic[Title/Abstract])) OR (Metabolic Cardiovascular Syndrome*[Title/Abstract]))) AND (("Prebiotics"[Mesh]) OR (((Prebiotic*[Title/Abstract]) OR (Probiotic*[Title/Abstract])) OR (Synbiotic*[Title/Abstract])))) AND ((((Placebo[Title/Abstract]) OR (Random*[Title/Abstract])) OR (trial[Title/Abstract])) OR (RCT*[Title/Abstract]))

# Supplementary Table

| **Table1** Characteristics of included trials. | | | | | | | | | | | | |
| --- | --- | --- | --- | --- | --- | --- | --- | --- | --- | --- | --- | --- |
| First author, Year | Country | Criteria for MetS | Sample  Size (Int/Con) | Age mean(year) | RCT design | Duration  (days) | Intervention | | Dietary interven-tion | Exercise interven-tion | Number of bacteria | Measured outcomes |
|  |  |  |  |  |  |  | Experimental group | Control group |  |  |  |  |
| Bellikci-Koyu 2019 | Turkey | IDF | 12/10 | Int:53.33  Con:52.67 | Parallel (Yes) | 84 | Kefir milk containing probiotics (180ml/day) | Regular milk | No | No | 6 | FBG、LDL-c、BMI、SBP |
| Bernini, 2016 | Brazil | ATP-III | 26/25 | NR | Parallel (Yes) | 45 | Milk containing probiotics（3.4×10^8^CFU） | Milk without probiotics | No | No | 2 | FBG、LDL-c、BMI、SBP |
| Carmen, 2019 | Spain | IDF | 25/28 | NR | Parallel (No) | 42 | Capsules containing probiotics（5×10^9^CFU） | Placebo | No | Yes | 1 | FBG、LDL-c、BMI、SBP |
| Cicero, 2020 | Italy | IDF | 30/30 | Int:72 Con:71 | Parallel (Yes) | 60 | Synbiotic vial（6×10^9^CFU）+ inulin and FOS | Placebo | Yes | Yes | 3 | FBG、LDL-c、BMI、SBP |
| Douglas, 2018 | Brazil | ATP-III | 23/22 | Int:47.0 Con:49.5 | Parallel (Yes) | 56 | synbiotic diet mousses（1×10^9^CFU）+4g mixture of inulin and fructooligosaccharide | Placebo diet mousses | No | No | 1 | FBG、LDL-c、BMI、SBP |
| **Table1** Continued | | | | | | | | | | | | |
| First author, Year | Country | Criteria for MetS | Sample  Size (Int/Con) | Age mean(year) | RCT design | Duration  (days) | Intervention | | Dietary interven-tion | Exercise interven-tion | Number of bacteria | Measured outcomes |
|  |  |  |  |  |  |  | Experimental group | Control group |  |  |  |  |
| Eslamparast, 2014 | Iran | ATP-III | 19/19 | Int:47.52 Con:46.05 | Parallel (Yes) | 196 | Synbiotic capsule (2×108CFU)+250mg fructooligosaccharide | Placebo | Yes | Yes | 7 | FBG、LDL-c |
| Mohammadi, 2018 | Iran | ATP-III | 44/43 | Int:45.4 Con:45.6 | Parallel (Yes) | 70 | Milk containing probiotics（1×107CFU）+3g inulin | Regular milk | No | No | 2 | FBG、LDL-c、BMI、SBP |
| Parastouei, 2020 | Iran | NR | 30/30 | Int:42.33 Con:40.6 | Parallel (Yes) | 56 | Synbiotic capsule（4.2×109CFU）+ fructooligosaccharide | Placebo | No | No | 7 | FBG、LDL-c、BMI、SBP |
| Rabiei, 2018 | Iran | NR | 20/20 | Int:57.1 Con:60.8 | Parallel (Yes) | 84 | Synbiotic capsule（2×108CFU）+125mg fructooligosaccharide | Placebo | Yes | No | 7 | FBG、LDL-c、BMI |
| Rahimi, 2022 | Iran | ATP-III | 52/56 | Int:42.77 Con:45.64 | Parallel (Yes) | 84 | Synbiotic capsule（1×109CFU）+38.5g FOS | Placebo | Yes | No | 7 | FBG、LDL-c、BMI、SBP |
| Rezazadeh, 2019 | Iran | NR | 22/22 | Int:44.05 Con:44.55 | Parallel (Yes) | 63 | Yogurt containing probiotics (4×106CFU) | Regular yogurt | No | No | 2 | FBG |
| Int: Intervention group; Con: Control Group; RCT: Randomized controlled trial; MetS: Metabolic syndromes; ATP-III:Adult Treatment Panel-III;IDF: International Diabetes Federation; CFU: Colony-forming unit; NR: Not reported; BMI: Body mass index; LDL-c: Low-density lipoprotein cholesterol; FBG: Fasting blood glucose; SBP: Systolic blood pressure. | | | | | | | | | | | | |
